# Diagnostic yield of CT-guided lung biopsies: how can we limit negative sampling?

**DOI:** 10.1259/bjr.20210434

**Published:** 2021-11-26

**Authors:** Marcello Andrea Tipaldi, Edoardo Ronconi, Miltiadis E Krokidis, Aleksejs Zolovkins, Gianluigi Orgera, Florindo Laurino, Julia Daffina, Damiano Caruso, Andrea Laghi, Michele Rossi

**Affiliations:** 1 Department of Surgical Medical Sciences and Translational Medicine, Sapienza University of Rome - Sant'Andrea University Hospital, Rome, Italy; 2 1st Department of Radiology, Aretaion University Hospital, Medical School, National and Kapodistrian University of Athens, Athens, Greece

## Abstract

**Objectives:**

To investigate whether lesion imaging features may condition the outcome of CT-guided lung biopsy (CTLB) and to develop a scoring system of biopsy outcome prediction.

**Methods:**

This is a single center retrospective study on 319 CTLBs that were performed in 319 patients (167 males/152 females, mean age 68 ± 12.2). Uni- and multivariate analysis were performed aiming to assess the imaging features that are likely to be correlated to a negative biopsy outcome and patients were stratified in groups accordingly.

**Results:**

Technical success was 100%. 78% of the biopsies (250/319) led to a concrete histology report (218 malignant/32 benign). The remaining lesions led to concrete histology at a second attempt that occurred on a later time. Multivariate analysis revealed increased risk of inconclusive result for nodules with low fludeoxyglucose uptake [odds ration (OR) = 2.64, 95% confidence interval (CI) 1.4–4.97; *p* = 0.003], for nodules with diameter smaller than 18 mm (OR = 2.03, 95% CI 1.14–3.62; *p* = 0.017) and for nodules that are located in one of the lung bases (OR = 1.96, 95% CI 1.06–3.62; *p* = 0.033). Three different groups of patients were identified accordingly with low (<30%), medium (30–50%) and high (>50%) probability of obtaining an inconclusive biopsy sample.

**Conclusion:**

This study confirms that percutaneous CT-guided biopsy in nodules that are either small in diameter or present low positron emission tomography-fludeoxyglucose uptake or are in one of the lung bases may lead to inconclusive histology. This information should be factored when planning percutaneous biopsies of such nodules in terms of patient informed consent and biopsy strategy.

**Advances in knowledge:**

Inconclusive histology after lung biopsy may be subject to factors irrelevant to technical success. Lung biopsy histology outcomes may be predicted and avoided after adequate planning.

## Introduction

Percutaneous CT-guided lung biopsy (CTLB) is a first-line, minimal invasive, image-guided procedure implemented for the histologic characterization of focal lung lesions.^1–3^ Lung cancer screening and increased use of CT led to a raise in the detection rate of lung masses; most of such masses would require further characterization as further management is not possible without a histological confirmation of pathology.^4–10^

CTLB is considered as a standard sampling technique, particularly for lesions located in a peripheral position in the lung parenchyma, allowing both histological and biomolecular analysis.^11^ However, in some cases, CTLBs may be inconclusive. There are some arbitrary criteria on which lesions might lead to a non-conclusive result when biopsied; however, there is no clear consensus given the heterogeneity of evidence in the literature.^12–16^

The aim of this study is to assess whether we may predict biopsy performance outcomes based on imaging features of the lesion. This information to our view is considered important in terms of following the appropriate biopsy strategy but also in terms of informed consent towards the patient that might need to undergo a repeated biopsy procedure.

## Methods and materials

### Patients

This is a single center retrospective study concerning the period between January 2014 and January 2019. Approval from the local ethics committee was obtained. We included in the study all the patients that underwent a CT-guided biopsy in our institution (Sant'Andrea University Hospital, La Sapienza, Rome, Italy) in the mentioned period and had in their records a fludeoxyglucose positron emission tomography (FDG PET) no longer than 6 months prior to the biopsy. Patients who underwent other procedures between the FDG PET and the biopsy were excluded. Out of the 1178 CTLBs that were performed in the mentioned period, 319 patients fulfilled the inclusion criteria and were included in the study.

### Biopsy procedure

All biopsies were performed by four interventional radiologists with more than 5 years of experience on percutaneous CT-guided interventions; a 16 slice (Mx8000 IDT, Philips Healthcare, Cleveland, OH, EE. UU) or a 256 (Brilliance ICT 256, Philips Healthcare, Cleveland, OH, EE. UU) multislice spiral scanner was used for the procedures.

Patients were placed in supine, prone or lateral positions according to the most suitable approach to the lesion and a low-dose helical CT examination was performed in order to plan the access. Needle pathway, when possible, was delineated, avoiding interlobular fissures, visible bronchi, and relatively large vessels. Necrotic areas of the lesions (based on CT and FDG PET findings) were not targeted.

During needle advancement, images were acquired intermittently using CT fluoroscopy, which allowed single acquisitions to confirm and adjust the needle position.^3^ Full-Core Coaxial needle systems 18/20 Gauge were used in all cases. One to four samples were obtained according to operators’ preference.

A second unenhanced low-dose helical CT performed at the end of the intervention to detect possible early complications.

### Data collection

Based on the obtained CT scan, lesion size, morphology and position including length of needle pathway from pleural surface to lesion were recorded. The latter was evaluated taking into account the optimal needle trajectory avoiding big size vessels, visible bronchi and interlobular fissures. A lesion was considered as located in one of the lung’s basal segments if a part of the diaphragm was included in the same CT frame as the lesion.

The standardized uptake value (SUV) was deducted from the PET report and an SUVmax equal or smaller to 2.5 was considered as the cut-off value for the purposes of this study, to consider a nodule as of low FDG PET uptake.

Data regarding each procedure have been extracted from the hospital patient records: total session duration, procedural time for biopsy and dose–length product (DLP) for each patient were collected. DLP was calculated considering the DLP of each scan and the number of scans performed in CT fluoroscopy.

Potential complications were assessed with chest plain films 3 h post-biopsy and when necessary, a CT scan.

### Definition of outcomes

Technical success was defined as the evidence of the needle tip in the target lesion and the achievement of material susceptible of histological evaluation.

Histopathology reports were classified in three categories: positive for a specific malignant disease, positive for a specific benign disease and not adequate. The latter were deemed those, which resulted in necrosis, clots, normal parenchyma tissue, or those with not enough material for diagnosis.

### Statistical analysis

Continuous variables were reported as mean (standard deviation, SD) or median (interquartile range, Q1–Q3). Distribution was checked for normality using the Shapiro–Wilk test. Categorical variables were presented as absolute number (n) and relative percentage frequency (%). To compare continuous variables between two groups, a two-tailed *t*-test was used when normally distributed; when the variables were not normally distributed, the non-parametric Mann–Whitney test was used. Pearson χ^2^ was performed to test the association between categories for dichotomous variables. The continuous variables, maximum lesion diameter and distance from the pleural surface were dichotomized around an optimal cut-off identified on receiver operating characteristic curves applying Youden’s index (the maximum value of sensitivity + specificity).

We considered not adequate outcome as the response variable and performed uni- and multivariable analyses applying a logistic regression model with cluster robust estimate of standard error, considering the repetition of some patients. The multivariable logistic model was built considering as independent predictors those which resulted significant at a level of significance of 0.10.

The results were reported in terms of odds ratio (OR) and relative 95% confidence interval (95% CI). The predictive accuracy of the model was assessed through the c-index and its confidence interval (95% CI); performing the Hosmer–Lemeshow test assessed the goodness-of-fit of the model. The Bootstrap resampling method was chosen for internal validation of predictive models by selecting 500 repetitions. For each group of 500 bootstrap samples, the model was reassembled and tested against the observed sample in order to estimate predictive accuracy and distortion. As regards to calibration, the bootstrap was used to obtain correct estimates (corrected for overfitting) of the expected values compared to the observed ones represented in the calibration graph. The multivariable logistic model has been represented through a nomogram, a graphic representation that traces the effect of each variable to a scale ranging from 0 to 100, in order to add the scores obtained by the variables in the model, on the basis of the values observed, and to have a total risk score that corresponds to a greater or lesser probability of a non-adequate outcome. A *p*-value < 0.05 was considered statistically significant. The software STATA 14.1 was used for all the statistical analyses.

## Results

### Demographic features, histological outcomes, and complications

Patients and nodules demographic features are reported in Table 1. Technical success was 100%. A concrete histological outcome was obtained in 78% (*n* = 250) of the 319 cases. There were 218 (87%) malignant and 32 (13%) confirmed benign lesions. Malignant lesions were mainly adenocarcinomas in 54% of the cases (*n* = 118), squamous cell carcinomas in 16% (*n* = 35) and metastatic lesions in 12% (*n* = 26). The rest of malignant histology was distributed among lymphomas (*n* = 9), non-differentiated tumors (*n* = 9), small cell carcinoma (*n* = 10), carcinoid (*n* = 5), mesothelioma (*n* = 4) and sarcoma (*n* = 2). Benign diagnosis included chronic inflammation and organized pneumonia (*n* = 14), necrotizing and non-necrotizing granulomatosis (*n* = 6), reactive lymphoid hyperplasia (*n* = 4), hamartochondromas (*n* = 4), solitary fibrous pleural tumor (*n* = 2) and lesions positive for the DNA of *Mycobacterium tubercolosis* (*n* = 2). Non-conclusive histology occurred in the 22% (*n* = 71) of patients due to inadequate samples.

**Table 1.** Patient and nodule demographic features

		Population *N* = **319**
**Age, years**	***Mean ± SD***	68 ± 12.2
	***Range***	32–88
**Sex**	***m***	*185*
	**f**	134
**Lesion diameter, mm**	***Median (IQR)***	23.25 (20.15)
	***Range***	5–120
**Distance from the pleural surface, mm**	***Median (IQR)***	14 (29)
	***Range***	0–75
**PET**	***Negative, n***	*70*
	***Positive, n***	249
**Basal localization**	***Yes, n***	75
	***No, n***	244
**Nodule aspect**	***Solid, n***	263
	***Subsolid, n***	40
	***GG’s, n***	16

IQR, interquartile range; PET, positron emission tomography; SD, standard deviation.

The mean session duration was 19 (±9 SD) min and the median duration of needle position and sampling was 8 min (interquartile range = 6–13). The median DLP value for the CT fluoroscopy time was 49.4 Gy x cm^2^ (interquartile range = 33.8–75.4).

Complications were classified according to the CIRSE Guidelines.^17^ In our population, we observed Grade 1 complications such as pneumothorax not requiring intervention, limited parenchymal hemorrhage and transient hemoptysis, Grade 2 complications such as pneumothorax or iatrogenic bleeding requiring patient observation with overnight stay, and Grade 3 complications such as major pneumothorax treated via, chest tube placement.

Overall complications rate was 45%, however in 40% of the cases complication was only Grade 1 (in 23% small pneumothorax that did not require any further intervention or prolonged hospital stay and in 22% small alveolar hemorrhage). In 5% pneumothorax that required the placement of drainage tube and prolonged hospital stay occurred (Grade 2–3).

### Nodule ^18^F-FDG-uptake and correlation with histology

The SUV max threshold of 2.5 was used as an arbitrary value to distinguish between PET “positive” (SUV max >2.5- Group A) and PET “negative” (SUV max ≤ .5 - Group B) nodules. In total, 250 (78.4%) lesions were included in Group A and 69 (21.6%) in Group B.

Conclusive histology outcomes were significantly higher for Group A (82% vs  61%, *p* = 0.0003) and radiation exposure was significantly higher for Group B (median DLP 66.3 mGy x cm *vs* 48.8 mGy x cm, *p* = 0.0008).

### Analysis of variables correlated with non-conclusive histology and predictive model

**Figure 1.**
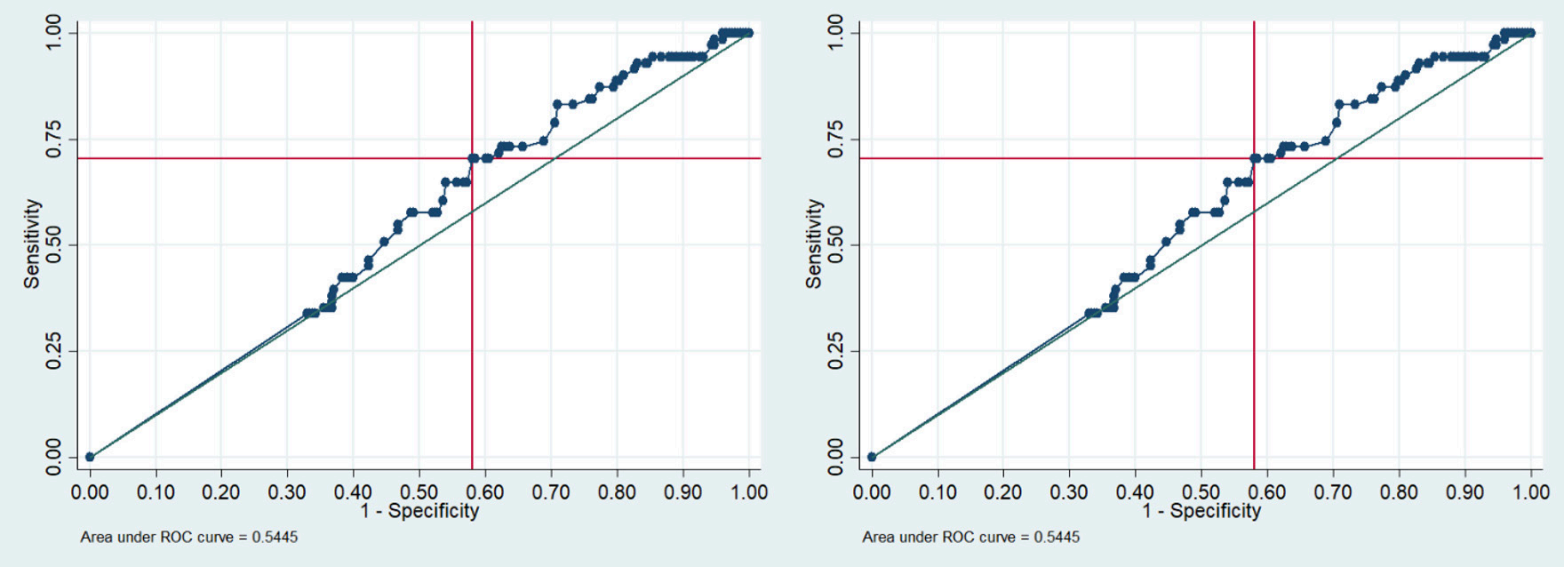
ROC curve for diameter (left side) and for distance from the pleural surface (right side). ROC, receiver operating characteristic.

Lesions were analyzed in terms of maximum diameter and distance from the pleural surface. A cut-off value of 18 mm was considered (after dichotomization of values) for maximum lesion diameter and of 20 mm for distance from the pleural surface (Figure 1 and Table 2).

**Table 2.** The continuous variables, maximum lesion diameter and distance from the pleural surface were dichotomized around an optimal cut-off identified on ROC curves applying Youden’s index

	Cut Off*	Sensitivity	Specificity	AUC	AUC 95% CI	
**Diameter, mm**	18.02	49.30%	68.15%	58.70%	52%	65.3%
**Distance from the pleural surface, mm**	20	70.42%	41.94%	56.2%	50%	52.3%

AUC, area under the curve; CI, confidence interval; ROC, receiver operating characteristic.

More specifically, a cut-off of 18 mm was identified for maximum lesion diameter and of 20 mm for distance from the pleural surface.

On the basis of the univariable analysis results, a multivariable logistic regression model was developed taking into account as independent variables the following: age at biopsy, FDG PET uptake, lesion localization in one of the lung bases, nodule diameter and distance from the pleural surface (Table 3). The results revealed a significantly higher adjusted probability of inconclusive sample for FDG PET negative nodules (OR = 2.64, 95% CI 1.4–4.97; *p* = 0.003), for nodules with diameter smaller than 18 mm (OR = 2.03, 95% CI 1.14–3.62; *p* = 0.017) and for nodules with localization in one of the two lung bases (OR = 1.96, 95% CI 1.06–3.62; *p* = 0.033). No significance was noted for the following parameters: (a) distance from the pleural surface smaller than 20 mm (1.65, 95% CI 0.9–3.03; *p* = 0.107) and (b) age at biopsy (OR = 0.98, 95% CI 0.97–1; *p* = 0.165).

**Table 3.** Uni- and multivariable logistic model results

		Univariable logistic analysis				Multivariable logistic analysis			
		*N* = **319**				*N* = **319**			
		OR	95% CI		p	OR	95% CI		p
Sex	F	1							
	M	0.93	0.55	1.58	0.794				
Age at biopsy	years	0.98	0.96	1	**0.093**	0.98	0.97	1	0.165
PET	Positive	1							
	Negative	3.29	1.83	5.92	**<0.001**	2.64	1.4	4.97	0.003
Nodule type	Solid	1							
	Subsolid	1.08	0.48	2.43	0.859				
	Groundglass	1.69	0.58	4.89	0.336				
	Yes	1.05	0.59	1.85	0.878				
Basal localization	No	1							
	Yes	2.1	1.19	3.69	**0.01**	1.96	1.06	3.62	0.033
Diameter	>18.02 mm	1							
	≤18.02 mm	2.08	1.22	3.56	**0.007**	2.03	1.14	3.62	0.017
Distance from the pleural surface	>20 mm	1							
	≤20 mm	1.72	0.97	3.06	**0.065**	1.65	0.9	3.03	0.107

PET, positron emission tomography.

**Figure 2.**
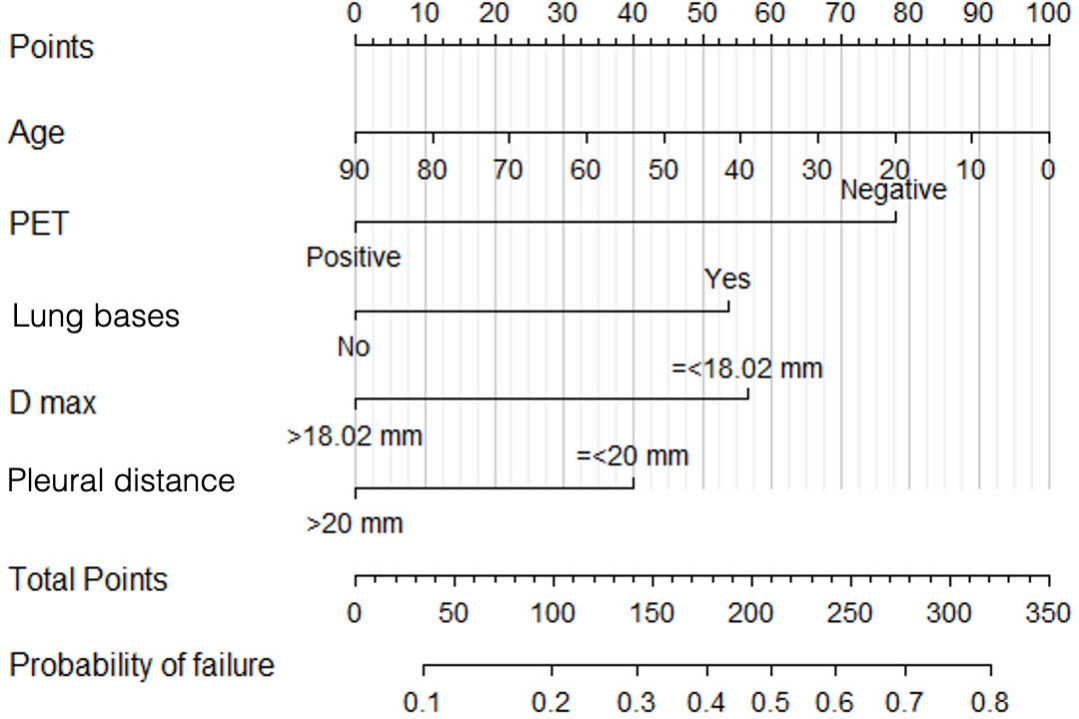
Nomogram. In the nomogram, each variable in the model was listed separately with a corresponding number of points assigned to a given magnitude of the variable. A total 78 points were allocated to PET, 57 points to nodule diameter, 54 points to basal localization, 40 points to pleural distance ≤20 mm, and an increasing number of points (0–100) the lower the patient’s age. The cumulative point score for all the variables was matched to a probability of occurring biopsy failure. Three groups of low (<30%), medium (30–50%) and high (>50%) risk of failure patients, were finally detected with corresponding cut-off points of <142, 142–210 and >210. PET, positron emission tomography.

**Figure 3.**
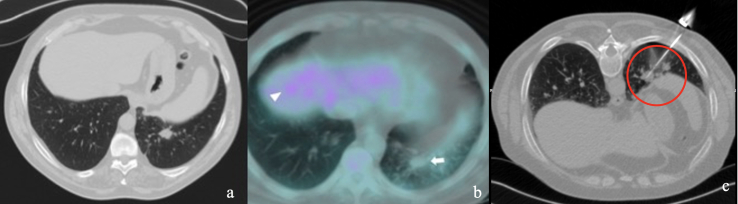
(a) High risk class - preprocedural CT shows a 16 mm solid nodule with basal localization in a 62-year-old patient. (**b**) ^18^FDG PET-CT of the nodule (white arrow) shows poor metabolic activity (SUVmax 2.4), noticeable when compared to the liver (white arrowhead). According to our scoring system, total points allocated for this lesion were 222 (33 for the patient age; 54 for localization in the lung basis; 57 for small dimension <18 mm; 78 for PET negative result). (**c**) Biopsy of the nodule shows needle tip at the centre of the target (red circle), yet collected material resulted not diagnostic due to scarcity of the material. ^18^FDG, 18-fludeoxyglucose; PET, positron emission tomography; SUV, standardized uptake value.

Multivariable model was graphically depicted by a nomogram (Figure 2), obtaining the probability of failure occurring for a given individual. In the nomogram, each variable in the model was listed separately with a corresponding number of points assigned to a given magnitude of the variable. A total of 78 points were allocated to PET, 57 points to nodule diameter, 54 points to basal localization, 40 points to pleural distance ≤20 mm, and an increasing number of points (0–100) the lower the patient’s age. The cumulative point score for all the variables was matched to a probability of occurring failure. Three groups of low (<30%), medium (30–50%) and high (>50%) risk of failure patients, were finally detected with corresponding cut-off points of <142, 142–210 and >210. A total of 262 patients were allocated in the low-risk group (83%), 45 in the medium risk group (14%) and 12 in the high-risk group (3%). The model reveals that the parameter “low FDG PET uptake” appears high correlation with inconclusive biopsy results. In our population, it was present in 100% of the high-risk group, in 75% of the medium group and in only 5% of the low-risk group. The other two parameters that appear correlated to histological outcome are the lesions diameter (smaller than 18 mm) and the localization of the lesion in one of the two lung bases that were present in 25, 70, 87% and 17, 39,73% of the patients in the respective risk groups. As an example, we can observe how a patient 62 years old (33 pts), with a PET negative nodule (78 pts), basal located (54 pts) with a diameter ≤18.02 mm (57 pts) had a total score of 222, corresponding to the high-risk group (Figure 3).

## Discussion

Percutaneous CT-guided biopsy of lung lesions is a well-established minimal invasive percutaneous procedure for the characterization of focal lung lesions. Even though success is high, and most lesions may be easily targeted under CT guidance in most cases, histology outcome is not always conclusive. A potential strategy for this issue may be the use of a larger diameter biopsy system or the increase of the number of obtained cores, however, this might come with the additional cost of higher complication rate such as pneumothorax.^18,19^ It is therefore very useful to be knowledgeable on which lesions are more likely to lead to a non-conclusive result when biopsied.

Our study was successful in identifying those intrinsic features of lung nodules that are more likely correlated with a biopsy failure. Overall success rate of CTLB performed in our cohort are comparable to those previously reported in literature, especially considering the mean lesion diameter of 2,3 cm in our cohort,^12–15^ with some degree of variability between groups. In particular, the three features that resulted significant in our study were the following: basal localization, dimension of the lesion and the ^18^F-FDG uptake.

Diameter of the lesion was found to be a determining factor in our series, and this is in line with the previous report of Huang at al^20^ who found the diagnostic accuracy of CTLB to be significantly lower in nodules <15 mm, as in other older series.^21,22^ However, other authors^23,24^ reported rare difference in the diagnostic accuracy of CTLB for small nodules such as less than 20 mm and 8 mm, respectively. In our opinion, there are several possible explanations for these findings: type of needle used and biopsy technique, as previously suggested by Laurent et al,^23^ differences in population selection and the definition of “successful” procedures could be implicated.

Another factor that may condition the outcome may be the number of samples for each lesion. In our study, the number of samples was collected and analyzed preliminary, but we did not find statistical correlation with success. However, due to non-rigorous documentation in the reports it wasn’t possible to collect these data for all the procedures and we decided not to include it in the final research.

Basal localization of the nodules is a well-known limiting factor of percutaneous lung biopsies given the vicinity with diaphragm and the inevitable mobility of the lesions due to the respiratory motion. In our series, it resulted significant in the univariate analysis with a value of *p* = 0.01 and in the multivariable logistic regression with a value of *p* = 0.033. Equally, lesion dimensions less than 18 mm resulted significant in the univariate analysis with a value of *p* = 0.007 and in the multivariable logistic regression with a value of *p* = 0.017.

Low ^18^F-FDG uptake was the factor, which was most significantly linked with inconclusive outcome (*p*=<0.001 in the univariate and *p* = 0.003 in the multivariate analysis). An explanation of this outcome may be the low cellular density present in some malignant lesions or the benign nature of the tissue, both factors correlated with a lower ^18^F-FDG uptake. Moreover, it’s well known that lung biopsies tend to perform worse in assessing benignity rather than malignancy.^8^

The predictive model was successful in identifying three groups of patients with different probability of failure respectively low, medium and high risk. Other two features were included to build the model: the distance from the pleural access less the 20 mm and the per year increment in age at biopsy.

The low-risk group account mostly PET positive nodules greater then 18 mm for which biopsy appears to be linked to a very low risk of failure (<30%). In our population, this group counted 262 patients with a concreate histological diagnosis of 81%.

The high-risk group, on the other hand, are mostly patients with PET-negative basal nodules, smaller than 18 mm and present a very high risk of failure (>50%). In our population, this group counted 12 patients with a concreate histological diagnosis of 44%. We recognize that this group of patients is, usually, low populated (12 cases in our series) and represents exceptional and demanding request to our service to reach a certain diagnosis.

**Figure 4.**
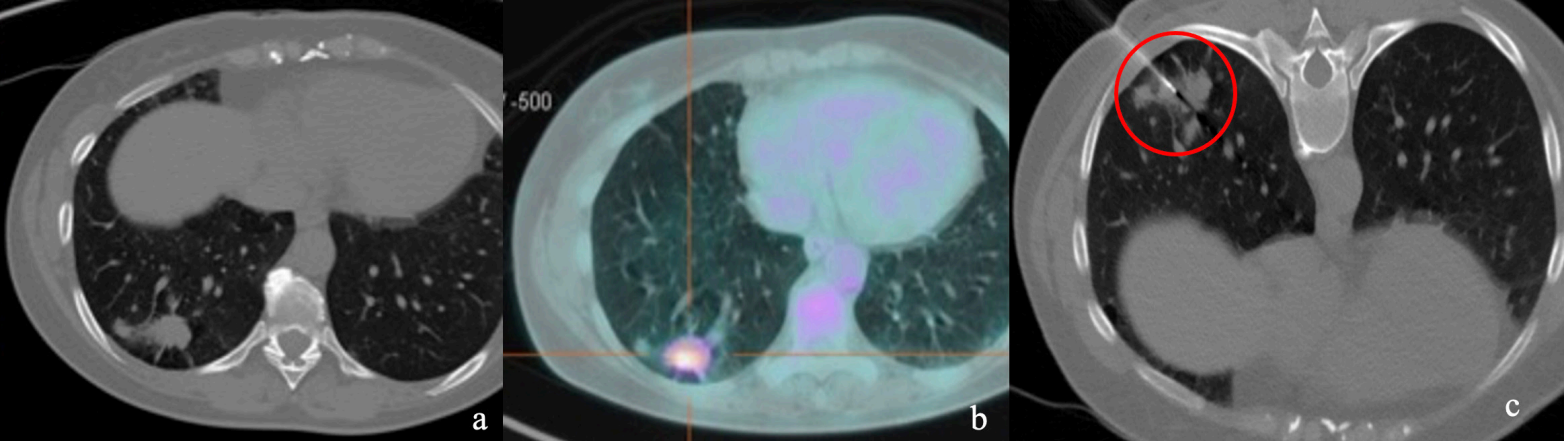
(a) Medium risk class - image shows a 24 mm solid nodule with a smaller satellite nodule located in the lower right lobe in a young patient (46 years old). (**b**) ^18^FDG PET-CT of the nodule highlights an increased metabolic activity (SUVmax 6.8). Total points allocated to this lesion were 150 (40 + 54+56). (**c**) Biopsy of the nodule revealed the malignant nature of the lesion (adenocarcinoma). ^18^FDG, 18-fludeoxyglucose; PET, positron emission tomography; SUV, standardized uptake value.

The group with medium failure risk (range between 30 and 50%) counted 45 patients in our series and presented a concreate histological diagnosis of 67%, which is quite low too (Figure 4). Even for these patients, we believe that CTLB should be carefully evaluated instead of other strategies. When applying the score to our population we can observe how the failure rate increased proportionally in the three groups (*p* = 0.005), as well as the procedure length and the delivered dose (*p* = 0.006; *p* < 0.001) (Table 4).

**Table 4.** Risk score applied in our population

	Low risk	Medium risk	High risk	*p*-value
**Outcome failure (%)**	19	33	56	*0.005*
**Procedure lenght (*min*) *Mean ± SD***	19 *±* 9	20 *±* 8	28 *±* 11	*0.006*
**Delivered dose *(DLP) Mean ± SD***	56.8 *±* 36.9	74.9 *±* 48.6	106.8 *±* 62.3	*<0.001*
**Complication rate**	43%	47%	67%	*0,678*

DLP, dose–length product; SD, standard deviation.

N.S.=Not significant at level 0.05.

Our study investigated which lesion imaging features condition the outcome of CTLB to minimalize the inconclusive procedures. Ceci et al^25^ recently reported how PET/CT-guided biopsy of lung lesions led to fewer inconclusive biopsies than CT-guided biopsy, confirming how the ^18^F-FDG uptake cover a key role in the potential histological characterization. Another recent report by Caruso et al^26^point out the ability of Radiomics CT-texture analysis in differentiating malignant from benign lung nodules with low FDG uptake, enhancing the importance of radiological heterogeneity beyond FDG PET/CT and lung biopsy. In this scenario, future studies are needed to assess if Radiomics features may give more information about the biopsy outcome prediction. The main limitation of our study is the lack of an external validation of the predictive model.

In conclusion, our study demonstrated how PET negative lung nodules are more difficult to characterize with percutaneous lung biopsy than PET-positive ones. Moreover, our predictive model has successfully identified two groups of patients with medium and high risk of biopsy failure where the procedure should be carefully evaluated, considering its invasive nature and the risk of longer procedure with higher rate of delivered radiation dose.
